# Systematic Annotation Reveals *CEP* Function in Tomato Root Development and Abiotic Stress Response

**DOI:** 10.3390/cells11192935

**Published:** 2022-09-20

**Authors:** Dan Liu, Zeping Shen, Keqing Zhuang, Ziwen Qiu, Huiming Deng, Qinglin Ke, Haoju Liu, Huibin Han

**Affiliations:** College of Bioscience and Bioengineering, Jiangxi Agricultural University, Nanchang 330045, China

**Keywords:** *Solanum lycopersicum*, CEP peptide, root growth, nitric oxide, nitrogen, salinity

## Abstract

Tomato (*Solanum lycopersicum*) is one of the most important vegetable crops worldwide; however, environmental stressors severely restrict tomato growth and yield. Therefore, it is of great interest to discover novel regulators to improve tomato growth and environmental stress adaptions. Here, we applied a comprehensive bioinformatics approach to identify putative tomato *C-TERMINALLY ENCODED PEPTIDE* (*CEP*) genes and to explore their potential physiological function in tomato root development and abiotic stress responses. A total of 17 tomato *CEP* genes were identified and grouped into two subgroups based on the similarity of CEP motifs. The public RNA-Seq data revealed that tomato *CEP* genes displayed a diverse expression pattern in tomato tissues. Additionally, *CEP* genes expression was differentially regulated by nitrate or ammonium status in roots and shoots, respectively. The differences in expression levels of *CEP* genes induced by nitrogen indicate a potential involvement of *CEP*s in tomato nitrogen acquisition. The synthetic CEP peptides promoted tomato primary root growth, which requires nitric oxide (NO) and calcium signaling. Furthermore, we also revealed that CEP peptides improved tomato root resistance to salinity. Overall, our work will contribute to provide novel genetic breeding strategies for tomato cultivation under adverse environments.

## 1. Introduction

Cell-to-cell communication is pivotal for plants to coordinate their growth in response to various developmental and environmental cues, and this cellular communication depends on small regulatory peptides [1,2]. Small secretory peptides are classified into two major groups based on their structure differences, the post-translationally modified peptide (PMT) or cysteine-rich polypeptides [3]. The C-TERMINALLY ENCODED PEPTIDE (CEP) peptide belongs to the PMT family, which often undergoes multiple proteolytic modifications. During this process, CEP peptides are cleaved into their bioactive form with approximately 15 amino acids in length [4,5,6]. *CEP* members have been identified across the plant kingdom; however, the biological function of most *CEP* genes remains largely untapped due to lack of loss-of-function mutants or genetic redundance [5,6,7,8,9,10,11,12,13,14,15].

In total, 15 *CEP* genes have been identified in model plant *Arabidopsis thaliana*. The *Arabidopsis CEP* genes are differentially expressed in various tissues, and they also respond to environmental stress, suggesting their diverse roles in the regulation of various aspects of *Arabidopsis* development and adaptions [5,6]. The application of synthesized *Arabidopsis* CEP1 (AtCEP1) peptide arrests root growth [4]. The synthesized AtCEP3 peptide inhibits primary root growth and lateral root emergence [5]. AtCEP5 is perceived by its putative receptor XYLEM INTERMIXED WITH PHLOEM 1 (XIP1)/CEP RECEPTOR 1 (CEPR1) to inhibit primary root growth and lateral root development [6,16]. These reports indicate crucial roles of *CEP*s in plant development.

Nitrogen is an essential macronutrient for plant growth and yield. CEP peptides have been reported to mediate nitrogen acquisition [17,18,19,20,21,22]. Under nitrogen starvation conditions, CEPR1 and CEPR2 receptors perceive and transmit the CEP peptides from roots to shoots, thus activating nitrate transporter gene expression, which resulted in nitrogen acquisition [17]. The shoot-derived polypeptides, CEP DOWNSTREAM 1 (CEPD1), CEPD2 and CEPD-LIKE2 (CEPDL2), act as descending long-distance mobile signals downstream of the CEP-CEPR pathway to activate the expression of nitrate transporter genes, thus mediating systemic nitrogen acquisition [18,19]. In *Medicago truncatula*, MtCEP together with its receptor COMPACT ROOT ARCHITECTURE 2 (MtCRA2) regulates nodulation numbers via modulating the expression of *NODULE INCEPTION* (*NIN*) transcription factor and *miR2111* [20,21,22], and also via ethylene and auxin hormone signaling [23]. Compared to nitrogen, *CEP* also regulates sucrose-dependent lateral root development [24]. The synthesized AtCEP3 peptide inhibits primary root growth by decreasing cell division under starvation conditions in a CEPR1-dependent manner [25]. Additionally, AtCEP5 plays roles in *Arabidopsis* osmotic and drought stress responses via interfering with auxin signaling [26]. These data suggest that *CEP*s play an essential role in plant environmental adaptions.

Tomato (*Solanum lycopersicum*) is one of major crop species with high economic value that is cultured world-wide, and there is a growing demand in the fresh fruit market and processed food industries. Notably, environmental stressors severely limit tomato growth and yield. Hence, the generation of tomato cultivars with enhanced growth and stress tolerance is one of the most sustainable approaches for its successful production [27]. The major goals of tomato breeding require identifying novel regulators of tomato development and stress adaptions from available genomic resources [28]. Here, we performed a systematic search of putative *CEP* genes in the tomato genome and then studied their potential physiological roles in tomato development and abiotic stress responses. We identified 17 tomato *CEP* genes in total, and they exhibited a diverse expression pattern in tomato tissues. In addition, we revealed that tomato *CEP* genes differentially responded to nitrogen status in roots and shoots, respectively. Exogenous application of the chemical synthesized tomato CEP peptides promoted tomato root growth, which requires nitric oxide (NO) and Ca^2 +^ signaling. Furthermore, the exogenous application of synthetic CEP peptides improved tomato root resistance to salinity stress. Taken together, our systematic study of the tomato *CEP* gene family demonstrates the potential physiological function of CEP peptides in tomato development and abiotic stress responses, and it will provide novel tactics for genetic breeding to improve tomato fitness and to increase the yield under adverse environmental conditions.

## 2. Results

### 2.1. Genome-Wide Annotation of Putative Tomato CEP Gene Family

**Figure 1:**
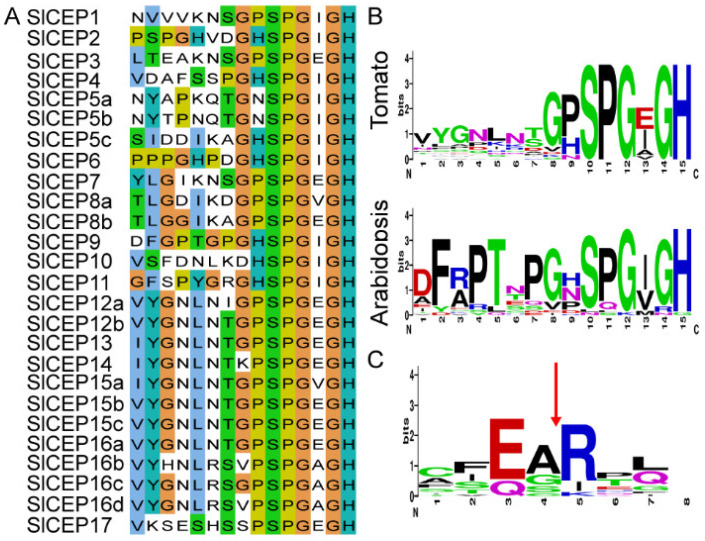
Identification of tomato *CEP* genes. (**A**) Alignment of CEP motifs of *SlCEP* genes. (**B**) Weblogo showing the consensus sequence of CEP motifs in tomato and *Arabidopsis*. (**C**) Weblogo showing the N-terminal signal peptide cleavage site of SlCEP proteins.

To determine the tomato *CEP* gene (named as *SlCEP*) family members, the previously reported 15 *Arabidopsis* CEP (AtCEP) and six tomato CEP full-length protein sequences were used as query sequences to perform protein BLAST searches against the re-assembly tomato genome (ITAG 4.0) [5,6,7]. As a result, a total of 17 *SlCEP* genes were identified (Figure 1A; Table S1). SlCEP proteins shared similar but divergent CEP motifs compared to the AtCEP proteins (Figure 1B and Figure S1) [5,6]. The *SlCEP* genes were re-arranged according to their location on the chromosomes (Table S1). The corresponding coding sequence (CDS) of *SlCEP*s ranged from 204 base pairs (*SlCEP13*) to 936 base pairs (*SlCEP17*) with the protein size ranging from 68 (*SlCEP13*) to 312 (*SlCEP17*) amino acids in length. The molecular weight and isoelectric point of SlCEP proteins ranged from 7365.5 Da (*SlCEP13*) to 32878.64 Da (*SlCEP17*) and from 6.58 (*SlCEP9*) to 9.92 (*SlCEP16*), respectively (Table S1). 

### 2.2. Motif Analysis, Gene Structure and Chromosome Localization of Slcep Proteins

The AtCEP proteins contain one or more conserved CEP motifs at the C-terminus [5,6]. Therefore, we analyzed the presence and distribution of CEP motifs in the 17 SlCEP proteins. Our analysis showed that SlCEP8 and SlCEP12 contained two CEP domains; SlCEP5 and SlCEP15 displayed three CEP domains; SlCEP16 showed four CEP motifs (Figure 1A; Table S1). Notably, some SlCEP proteins shared identical CEP motifs (Figure 1A). The N-terminal signal peptide proteolytic processing is essential for generating mature and functional CEP peptides [29,30]; we next searched for the presence and location of the putative N-terminal signal peptide cleavage sites in each SlCEP proteins. Based on the prediction, it is likely that the cleavage site occurs at a conserved arginine site (Figure 1C; Table S2), which has been also shown in CEP proteins identified in other plant species [10,12,13]. However, we did not find any cleavage site for SlCEP12; this may be due to the limitations of the software.

**Figure 2:**
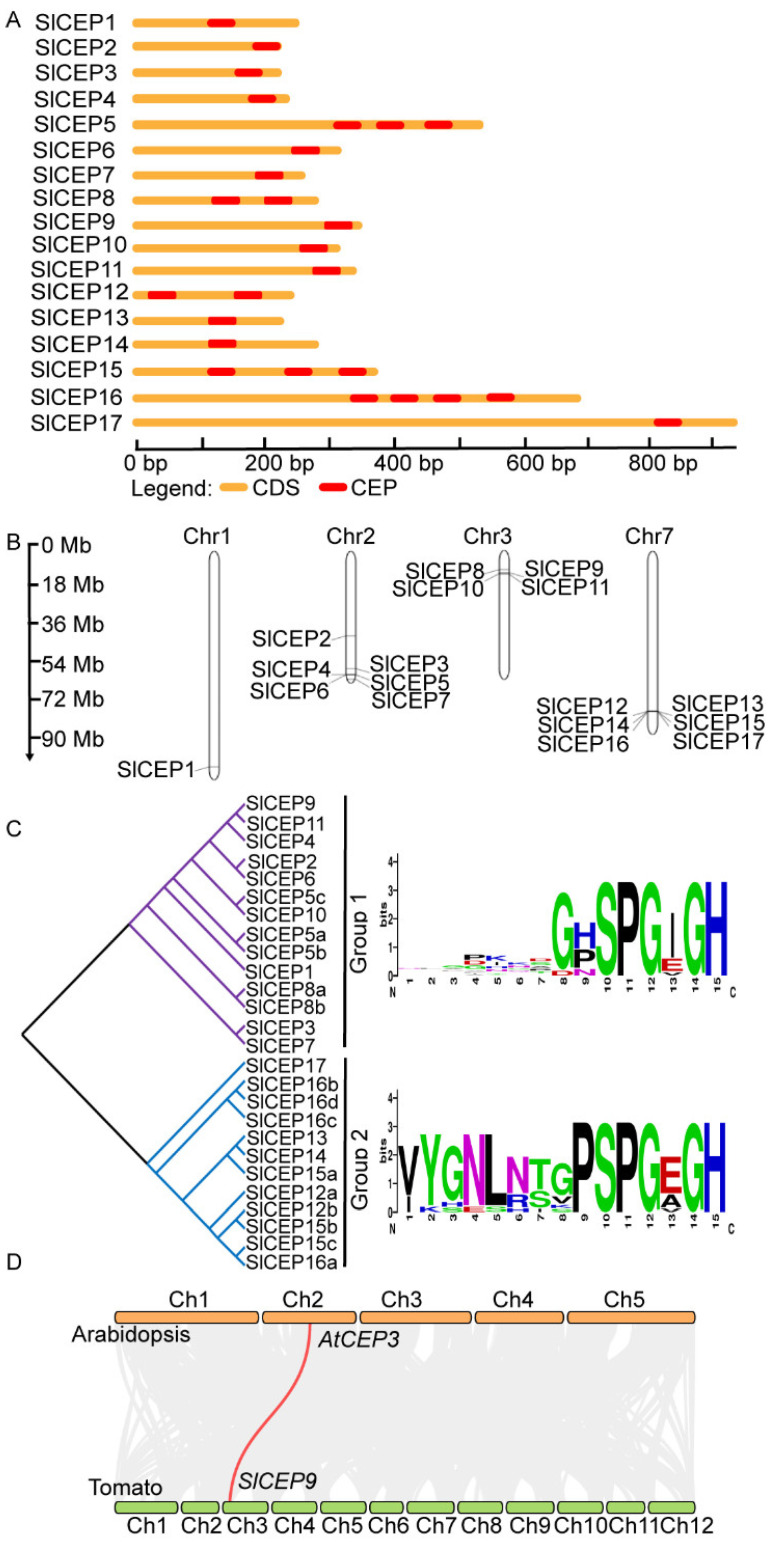
Genomic organization and chromosome localization, and phylogenetic analysis of *SlCEP* genes. (**A**) Gene structure of *SlCEP* genes. (**B**) Distribution of *SlCEP* genes on tomato chromosomes. (**C**) SlCEP proteins are classified into two major groups based on the CEP motifs. Weblogo showing the consensus sequence of CEP motifs in each subgroup. The phylogenetic tree was generated based on the CEP motifs of SlCEP proteins with 1000 bootstrap replicates. (**D**) The gene duplication analysis of the *CEP* genes of *A. thaliana* with *S. lycopersicum*. The gray lines (in the background) represent collinear blocks between the respective genomes. The red lines indicate the syntenic gene pairs of *S. lycopersicum* with *A. thaliana*.

Gene structural analysis of the *SlCEP*s showed that *SlCEP*s lacked introns (Figure 2A). Analysis of the chromosomal location showed that 17 *SlCEP*s were mapped on four chromosomes (Chr1, Chr2, Chr3, Chr7) at different densities (Figure 2B). For example, *SlCEP4*, *SlCEP5*, *SlCEP6* and *SlCEP7* were organized sequentially in tandem on chromosome 2. A similar cluster was also observed for *SlCEP8*, *SlCEP9*, *SlCEP10* and *SlCEP11* on chromosome 3; and it was observed for *SlCEP12*, *SlCEP13*, *SlCEP14*, *SlCEP15*, *SlCEP16* and *SlCEP17* on chromosome 7. Notably, the clustered SlCEP proteins showed low sequence similarity but shared the consensus CEP motifs, suggesting that these genes might arise from recent tandem duplication events.

### 2.3. Phylogenetic Analysis of SlCEP Proteins

To further gain insights into evolutionary relationships among *SlCEP*s and to group them within the established subfamilies, SlCEP and AtCEP proteins were used to construct an unrooted phylogenetic tree. The SlCEPs were divided into two subgroups based on the CEP motifs (Figure 2C), and the CEP motifs of the two groups were aligned, resulting in a consensus of sequences supporting the classification of the two groups (Figure 2C). Notably, the phylogenetic relationship based on the CEP domains was not well supported when the full-length sequences of SlCEP proteins were analyzed, which was due to the amino acids outside the CEP motifs (Figure S2).

The phylogenetic relationship between AtCEP and SlCEP proteins was also analyzed using either the conversed CEP motifs (Figure S3) or the full-length protein sequences (Figure S4). The SlCEP and AtCEP proteins were grouped into several clades with varying degrees (Figures S3 and S4). We further investigated the evolution and origin of the *CEP* genes of tomato in comparison with *Arabidopsis* (Figure 2D). We only identified one pair of the syntenic relationship between *Arabidopsis* and tomato, where *SlCEP9* was linked to *AtCEP3*; this may suggest a distant evolutionary relationship between these two species. However, the tandem gene duplication events may occur within the tomato genome, as *SlCEP12*, *SlCEP15* and *SlCEP16* shared complete identical CEP motifs (Figure 1A). The divergent evolution of *SlCEP* genes suggested that SlCEP peptides may play divergent roles in tomato development compared to the well-known AtCEP peptides.

### 2.4. Distinct Expression Pattern of SlCEP Genes in Response to Developmental and Nitrogen Signal

The spatial gene expression patterns are essential for investigating gene function in various developmental processes; hence, we searched the public tomato transcriptome database to explore the *SlCEP*s expression patterns in tomato tissues. The *SlCEP* genes’ expression levels in 12 tissues were visualized (Figure S5). The expression patterns of *SlCEP*s were varied in the examined tissues. For example, *SlCEP7*, *SlCEP8*, and *SlCEP10* showed a relative high expression level in roots; *SlCEP16* was highly expressed in fruit, and *SlCEP2* was highly expressed in young flower buds, indicating their potential role in regulating various tomato developmental processes.

**Figure 3:**
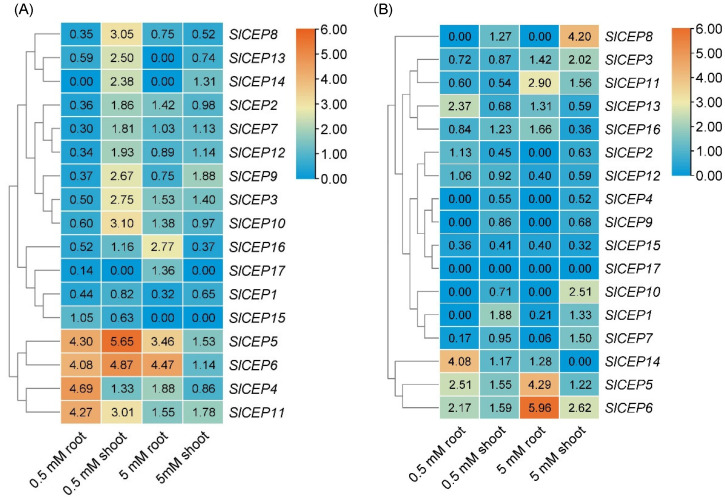
*SlCEP* genes expression are differentially regulated by nitrogen status in tomato roots and shoots. Expression levels of *SlCEP* genes in roots and shoots were quantified under low (0.5 mM) and high (5 mM) nitrate (**A**) and ammonium (**B**) treatment for 72 h. Relative expression levels of the genes were normalized to normal nitrogen condition, and the color represents log_2_ values. The heatmap was generated by TBtools.

Nitrogen regulates *AtCEP* genes expression; then, CEPR receptors sense the CEP signal to activate nitrogen transporter genes transcriptions, which resulted in nitrogen acquisition [17,18,19]. Plants use either nitrate (NO_3_^−^) or ammonium (NH_4_^+^) as a nitrogen resource; we then examined the expression profiles of *SlCEP*s under nitrate or ammonium treatment. The tomato seedlings were treated with low (0.5 mM) and high (5 mM) nitrate or ammonium for 72 h, and the roots and shoots, respectively, were collected for gene expression analysis. Under low nitrate treatment, *SlCEP4*, *SlCEP5*, *SlCEP6*, and *SlCEP11* were significantly upregulated, and *SlCEP1*, *SlCEP2*, *SlCEP3*, *SlCEP7*, *SlCEP8*, *SlCEP9*, *SlCEP12*, *SlCEP16 SlCEP17* were downregulated in roots; *SlCEP3*, *SlCEP5*, *SlCEP6*, *SlCEP8*, *SlCEP9*, *SlCEP10*, *SlCEP11*, *SlCEP13*, and *SlCEP14* were greatly upregulated in shoots. Under high nitrate treatment, *SlCEP5*, *SlCEP6*, *SlCEP16* were greatly upregulated and *SlCEP1* was downregulated in roots; and *SlCEP8* and *SlCEP16* were prominently downregulated in shoots. Under low ammonium treatment, *SlCEP5*, *SlCEP6*, *SlCEP13*, and *SlCEP14* were significantly upregulated and *SlCEP15* was downregulated in roots, while *SlCEP2* and *SlCEP15* were prominently downregulated in shoots. Under high ammonium treatment, *SlCEP5*, *SlCEP6*, and *SlCEP11* were upregulated and *SlCEP1*, *SlCEP7*, *SlCEP12*, *SlCEP15* were downregulated in roots; *SlCEP3*, *SlCEP6*, *SlCEP8,* and *SlCEP10* were upregulated and *SlCEP15* and *SlCEP16* were downregulated in shoots. Our analysis indicated that the expression levels of *SlCEP*s in roots and shoots varied greatly among nitrate or ammonium treatment, implying crucial but divergent roles of *SlCEP*s in tomato nitrogen acquisition (Figure 3). Considering some *SlCEP*s were not detected under our experimental conditions, this may be due to their spatio-temporal expression patterns or their responses to a certain nitrogen form and status. Overall, our analysis reveals the expression pattern of *SlCEP*s in response to nitrate or ammonium status, and *SlCEP*s would play diverse roles in nitrogen acquisition by regulating the corresponding nitrogen transporters in tomato roots and shoots, respectively (Figure 3) [17,18,19].

### 2.5. NO and Ca^2+^ Mediate CEP Peptide to Promote Tomato Root Growth

**Figure 4:**
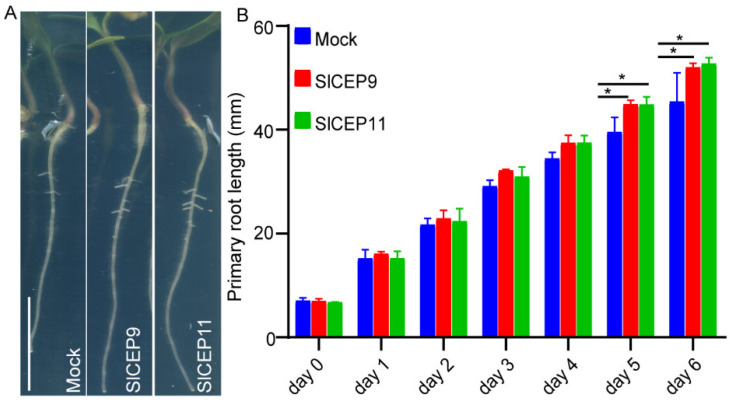
SlCEP9 and SlCEP11 peptide promote tomato root growth. (**A**) Representative images showing the SlCEP9 and SlCEP11 peptide-treated tomato primary root for 6 days. (**B**) Quantification of tomato primary root length upon synthetic SlCEP9 and SlCEP11 peptide treatment for 6 days. *n* = 10–15 seedlings, data represent mean ± SD, * *p* < 0.05 was determined by one-way ANOVA. Scale bar = 1 cm.

Next, we synthesized SlCEP9 and SlCEP11 peptides, the most identical CEP peptides to known function of AtCEP (Figure 2D and Figure S1), as examples to verify the physiological function of SlCEP peptides in tomato root development. After germination, tomato seedlings with a similar primary root length were transferred to new plates supplied with the synthetic SlCEP peptides and were cultured for another 6 days. Under our experimental conditions, we observed that tomato seedlings treated with both SlCEP9 and SlCEP11 peptide showed a significantly longer primary root (Figure 4).

**Figure 5:**
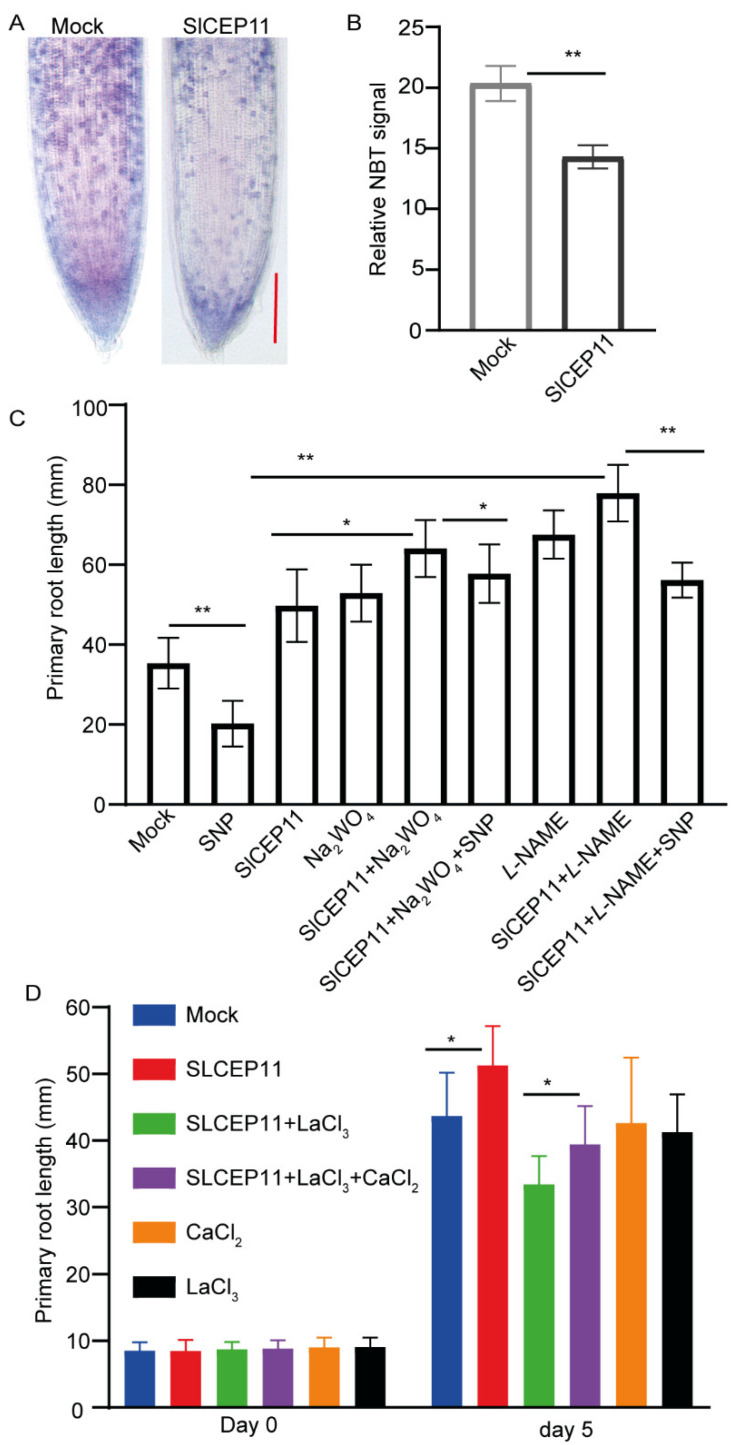
NO and Ca^2+^ are required for SlCEP11-mediated tomato root growth. (**A**) Representative images showing the NBT staining in synthetic SlCEP11 peptide treated tomato primary root for 6 days. (**B**) Quantification of NBT signal intensity. (**C**) Quantification of tomato primary root length upon NO inhibitors treatment in presence of synthetic SlCEP11 peptide for 6 days. (**D**) Quantification of tomato primary root length upon Ca^2+^ inhibitor treatment in presence of synthetic SlCEP11 peptide for 6 days. *n* = 10–15 seedlings, data represent mean ± SD, * *p* < 0.05 and ** *p* < 0.01 were determined by one-way ANOVA. Scale bar = 200 μm.

It has been reported that the inhibition of NO could promote tomato root growth [31,32,33]; we then focused on the involvement of NO in SlCEP11-mediated tomato root growth. NO can be oxidized to NO_2_, and superoxide can be detected by NBT staining; hence, we performed NBT staining as an indirect indication of NO level in SlCEP11 peptide-treated tomato primary roots. Our staining result revealed that SlCEP11 peptide significantly reduced the superoxide level compared to control treatment (Figure 5A,B), suggesting a potential reduced NO level triggered by the SlCEP11 peptide. We next assessed the effect of NO inhibitors on SlCEP11 peptide-mediated root growth. In line with previous reports [31,32,33], tomato primary root growth was promoted when NO signaling was inhibited and SNP (a NO donor) repressed the primary root growth. However, SlCEP11 and NO inhibitor exhibited a synergistic effect on primary root growth (Figure 5C). When exogenous NO was supplied, the synergistic effect was partially abolished, suggesting that NO is involved in SlCEP11 peptide function (Figure 5C). Ca^2+^ is an important signaling for plant development [34]; we then addressed whether Ca^2+^ participates in SlCEP11-mediated root growth. When the calcium channel was blocked by LaCl_3_, root growth promotion triggered by the SlCEP11 peptide was also counteracted (Figure 5D), and exogenous Ca^2+^ partially suppressed the LaCl_3_ effect. These data indicate an involvement of calcium signaling in SlCEP11-mediated root growth. Taken together, these preliminary data showed that NO and Ca^2+^ were involved in SlCEP peptide function in tomato root development.

### 2.6. SlCEP Peptide Promotes Tomato Root Resistance to Salinity

**Figure 6:**
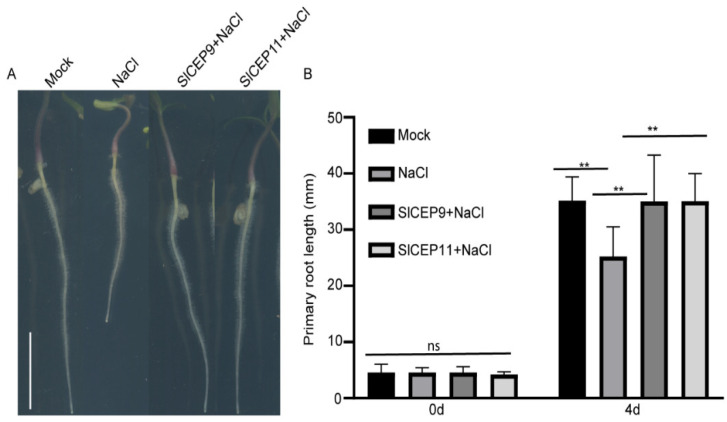
SlCEP9 and SlCEP11 promote tomato root growth under NaCl treatment. (**A**) Representative images showing salinity treated tomato primary roots for 4 days. (**B**) Quantification of tomato primary root length upon synthetic SlCEP9 and SlCEP11 peptide treatment in presence of 100 mM NaCl for 4 days. *n* = 10–15 seedlings, data represent mean ± SD, ** *p* < 0.01 was determined by one-way ANOVA. Scale bar = 1 cm. ns: no significance.

The CEP peptide has also been suggested to play roles in stress response [5,11,26]. The *atcep3* mutant displayed resistance to slat stress [5]; hence, we investigated whether the synthetic SlCEP9 and SlCEP11 peptides play a role in salinity response. After germination, tomato seedlings with a similar primary root length were transferred to new plates supplied with the synthetic SlCEP peptides in the presence of 100 mM NaCl and were cultured for another 4 days. Under our experimental conditions, we observed that salinity stress greatly inhibited tomato primary root growth; however, tomato seedlings treated with synthetic SlCEP peptides displayed longer primary roots, implying that SlCEP pep could improve tomato fitness under salt stress (Figure 6).

## 3. Discussion

**Figure 7:**
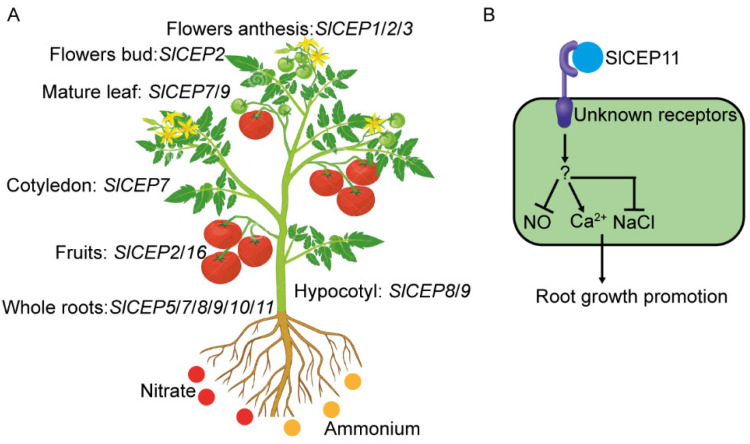
Summary of our work. (**A**) Expression pattern of *SlCEP*s in tomato tissues based on the public RNA-seq data. *SlCEP*s were also differentially regulated by nitrate and ammonium in roots and shoots, respectively. (**B**) A proposed working module for SlCEP11 peptide in tomato root development. SlCEP11 was perceived by unknown receptors, then regulated an undefined player to modulate NO, Ca^2+^ and salinity signaling, ultimately leading to root growth promotion.

Tomato is an essential cultural crop; however, the underlying mechanisms for tomato growth and development remain elusive. Numbers of studies have been reported that the CEP peptide family plays crucial roles in a wide range of plant developmental processes [29]. The CEP peptide family has been identified across various plant genomes; however, little is known about this family in tomato. Hence, we performed a genome-wide searching of putative tomato CEP peptide family to explore their potential physiological function in tomato development and stress responses (Figure 7).

To address the physiological roles the *SlCEP* genes, publicly available RNA-seq data were extracted to study the expression patterns of *SlCEP* genes in tomato tissues (Figure S5). The *SlCEP* gene family displayed diverse expression patterns, suggesting the diverse roles of *SlCEP*s in controlling various aspects of tomato development. Considering that only some of the *SlCEP*s can be detected in examined tissues, the expression patterns of these undetectable *SlCEP*s require further investigation in the future.

Exogenous application of synthetic CEP peptides regulates root growth and development, which mimics its endogenous functions [29]. Our data showed that the application synthetic SlCEP peptides promoted tomato primary root growth (Figure 4). Notably, the promotion of tomato root growth triggered by the exogenous application of synthesized SlCEP peptides just suggests their potential physiological roles and by no means limits their function in other developmental processes or rules out other SlCEP peptides as pivotal regulators in tomato growth and stress responses. Compared to the well-known inhibitory roles of the AtCEP peptides [4,5,16,29], the CEP peptides in tomato, cucumber and Brassica rapa exhibited an oppositive effect (Figure 4) [12,13]. Antagonistic peptide technology has been proposed to re-write CLE peptide function [35,36], the promotion of primary root growth triggered by SlCEP peptides (Figure 4), indicating that SlCEP9 and SlCEP11 may be an antagonistic form. However, it definitely requires careful examinations. On the other hand, it is likely that different downstream regulatory networks could be activated by SlCEP peptides in tomato roots. It is also necessary to investigate whether conserved serine (at position 10) and glycine (at position 14) are crucial for SlCEP peptide function, as these amino acid residues are important for apple MdCEP1 function [10]. The loss-of-function or gain-of-function of *SlCEP* mutants would assist with better elucidating the untapped physiological functions in the tomato life cycle. In addition, the CEP peptide is perceived by membrane localized CEPR receptors to trigger downstream responses [29]. A homology of AtCEPR receptors has been identified in tomato, and it can recognize CEP peptides [37]. It is interesting to test whether the tomato CEPR receptor can transmit the SlCEP signal to regulate tomato root growth and responses to environmental cues. On the other hand, 234 LRR-RLK receptors genes have been identified in tomato [38], suggesting various combination of SlCEPs and SlRLKs to modulate tomato development and environmental adaptions. Screening CRISPR-Cas9 targeted tomato receptor mutants [39] will assist with identifying novel corresponding receptors for SlCEP peptides. Additionally, the usage of 4-azi-dosalicylic acid ([^125^ I] ASA)-labeled SlCEP peptides could assist with screening the RLK library in BY2 cells, and it will also help identify their putative binding proteins [40].

Plants are unable to adjust their growth in the ever-changing environments when reactive oxygen species (ROS) homeostasis is disturbed in the roots, indicating the essential role of ROS in root development [41,42]. We showed that NO is required for SlCEP peptide function in tomato root development (Figure 5A–C). How SlCEP peptides regulate NO biosynthesis and metabolism or proteins which are main targets of NO-related post-translational modifications [43] requires further investigations. Additionally, it is also necessary to detect endogenous NO levels using a DAF-FM-DA probe [44]. Hydrogen peroxide (H_2_O_2_) is also involved in CEP-mediated root growth [12,13]; it is intriguing to test the involvement of H_2_O_2_ in SlCEP-mediated tomato root growth. Additionally, Ca^2+^ was also involved in SlCEP-mediated root growth (Figure 5D). The genetic mutants related to ROS biosynthesis and signaling mutants, as well as Ca^2+^ signaling mutants [45,46,47,48], will help to corroborate the critical roles of ROS and Ca^2+^ in the SlCEP peptide signaling pathway.

Abiotic stress negatively affects plant growth and productivity. Therefore, plants have evolved multiple mechanisms such as an increased expression of the stress-associated genes or hormones level to control their adaption to the ever-changing environments [49]. The small peptide family works in parallel with plant hormones to regulate plant stress responses [11,20,26]. Our qRT-PCR analysis showed that *SlCEP*s expression levels in roots and shoots were differentially regulated by nitrate or ammonium status, suggesting the potential involvement of *SlCEP*s in modulating tomato adaptions to the nitrogen status (Figure 3). In the past few decades, the nitrogen signaling regulatory networks have been established, and many genes play crucial roles in modulating nitrogen acquisition [50]; how SlCEP peptides recruit these known or undefined nitrogen regulators in tomato nitrogen adaptions needs to be determined in future investigations. Furthermore, the exogenous application of SlCEP peptides promoted tomato root resistance to salinity (Figure 6). However, the mechanisms underlying SlCEP-mediated salt stress responses requires more investigations [49]. Additionally, it is also worthwhile to reveal the untapped functions of *SlCEP*s in other developmental processes as well as abiotic and biotic stress responses.

## 4. Materials and Methods

### 4.1. Genome-Wide Annotation of Tomato CEP Peptide Family

In total, 15 *Arabidopsis* and 6 previously identified tomato CEP proteins [5,6,7] were used to perform protein BLAST searches against a re-assembled tomato genome (ITAG 4.0) released to Phytozome 13 (https://phytozome-next.jgi.doe.gov/, V13, accessed on 16 March 2022) [51]. Each newly identified protein was subsequently used to conduct protein blast against the tomato genome to avoid any missed SlCEP proteins until no novel proteins were found.

### 4.2. CEP Motif Analysis

Motif Alignment & Search Tool (MAST) and Find Individual Motif Occurences (FIMO) analyses (https://meme-suite.org/meme/, Version 5.4.1, accessed on 28 March 2022) [52] were performed to further clarify the CEP domains in all identified proteins, and proteins with a similar CEP domain were defined as SlCEP peptides [5,6,7]. SlCEP domain features were determined by Weblogo 3 (http://weblogo.berkeley.edu/logo.cgi/, Version 3.7, accessed on 17 April 2022) [53].

### 4.3. SlCEP Protein Features Analysis

SlCEP protein N-terminal signal peptide prediction was performed by searching SignalP 5.0 (http://www.cbs.dtu.dk/services/SignalP/, accessed on 30 April 2022) and Signal-CF (http://www.csbio.sjtu.edu.cn/bioinf/Signal-CF/, accessed on 30 April 2022) websites. The ExPASy Proteomics Server tool (https://web.expasy.org/compute_pi/, accessed on 20 April 2022) was used to analyze the theoretical average protein isoelectric point (pI) and molecular weight (MW) of SlCEP proteins [54].

### 4.4. Genomic Organization and Chromosome Localization

The genomic sequences and corresponding coding sequences (CDS) of the 17 *SlCEP* genes were downloaded from Phytozome 13. The genomic organization of the *SlCEP* genes was presented via a gene structure display server (http://gsds.cbi.pku.edu.cn, Version 2.0, accessed on 6 May 2022) [55]. MG2C online software (http://mg2c.iask.in/mg2c_v2.0/, Version 2.0, accessed on 8 May 2022) was used to analyze *SlCEP* genes distribution on chromosomes [56].

### 4.5. Alignment and Phylogenetic Analysis

ClustalX was applied for multiple alignment analysis [57]; the alignments were then refined and displayed via Jalview [58]. MEGA X software (https://www.megasoftware.net/, Version 10, accessed on 13 May 2022, and the software was downloaded and installed) was used to build the phylogenetic trees [59] using the conserved CEP domains or the full length of CEP proteins by the neighbor-joining method. Bootstrap analysis was conducted with 1000 replicates to verify the significance of nodes.

### 4.6. Gene Duplication Analysis

The *Arabidopsis thaliana* (TAIR 10) and *Solanum lycopersicum* (ITAG 4.0) genome and annotation files were downloaded from the Phytozome website. TBtools was used to scan the genome to identify duplicated gene pairs. Finally, the orthologous gene pairs were identified using a Dual synteny plotter in TBtools (https://github.com/CJ-Chen/TBtools/releases, Version 1.0987663, accessed on 16 May 2022, and the software was downloaded and installed) [60].

### 4.7. SlCEP Gene Expression in Tomato Tissues

A published tomato RNA-seq data in wild species *S. pimpinellifolium* (LA1589) was used to determine the expression patterns of the *SlCEP* genes in various tomato tissues (D006, http://ted.bti.cornell.edu/, accessed on 25 May 2022).

### 4.8. Plant Material and Growth Conditions

The cultivar tomato seeds “Dahong” were brought from Shanghai Hongqiao Tianlong Seed Company and were used in this study. All seeds were washed with distilled water. Tomato seeds were sterilized with 2.3% sodium hypochlorite for 5 min. The sterilized seeds were washed with distilled water 5–6 times. The seeds were kept in darkness at 28 °C to induce germination. The seedlings were grown in a plant growth chamber (16 h light: 8 h dark photoperiod, 21 °C, 112 μmol m^−2^ sec^−1^).

### 4.9. Total RNA Extraction and Gene Expression Analysis

After germination, tomato seedlings with a similar root length were transferred to liquid 1/2 MS solution (normal nitrogen as control) or liquid Hoagland solution without nitrogen (NS10205-NCoolaber, China) supplied with KNO_3_ (0.5 and 5 mM) or NH_4_Cl (0.5 and 5 mM) for 72 h, and the roots and shoots parts were collected, respectively. The RNA extraction kit (DP432, Tiangen, China) was used to extract total RNA. A Hifair III cDNA synthesis kit was used to generate first-strand cDNA from 1 μg of total RNA (Cat NO. 11139ES60, Yeasen Biotechnology, Bejing, China). The qRT-PCR was performed using Hieff qPCR SYBR Mix (Cat NO. 11170ES03, Yeasen Biotechnology, China) with an ABI 7500 Real-Time PCR System (Thermo Fisher, Waltham, MA, USA). The primers used for qRT-PCR analysis are listed in Table S3. The average expression level of SlCEP genes was calculated using the ^ΔΔ^CT method via TBtools [60,61]. Three independent experiments were performed.

### 4.10. SlCEP Peptides Treatment

SlCEP9 (DFGPTGPGHSPGIGH) and SlCEP11 (GFSPYGRGHSPGIGH) were synthesized by DGpeptide company. All peptides were dissolved in distilled water to a concentration of 10 mM and were stored at −20 °C. After germination, tomato seedlings with a similar primary root length were transferred to new plates supplied with 1 μM of SlCEP9 and SlCEP11 peptides, respectively. Plates were imaged via the EPSON V370 scanner. Primary root length was quantified via ImageJ software. Three independent experiments were performed.

### 4.11. NBT (Nitroblue Tetrazolium) Staining

After germination, tomato seedlings with a similar root length were transferred to new agar plates supplied with 1 μM of SlCEP11 peptide, and the seedlings were cultured for another 6 days; then, the seedlings were used for NBT staining. The seedlings were incubated in NBT staining buffer (0.5 mg/mL NBT in 50 mM phosphate buffer pH = 7.6) for 5 min in the dark. An inverted BDS 400 microscopy was used to capture the pictures. The relative NBT signal was quantified via ImageJ. Three independent biological repeats were performed.

### 4.12. NO and Ca^2+^ Inhibitor Treatment

After germination, tomato seedlings with a similar root length were transferred to new agar plates supplied with NO inhibitors (L-NAME, 25 μM, NO synthase-like enzyme inhibitor and Na_2_WO_4_, 5 μM, nitrate reductase inhibitor), SNP (50 μM, a NO donor), lanthanum chloride (LaCl_3_, 500 μM, Ca^2+^ channel blocker), and CaCl_2_ (500 μM) in the presence of 1 μM of SlCEP11 peptide for another 6 days. Plates were imaged via the EPSON V370 scanner. The root length was quantified via ImageJ. Three independent biological repeats were performed.

### 4.13. Salinity Treatment and Root Growth Quantification

After germination, tomato seedlings with a similar primary root length were transferred to new agar plates supplied with 100 mM NaCl in presence of 1 μM of SlCEP9 and SlCEP11 peptide, respectively, and the seedlings were cultured for another 4 days. Plates were imaged via the EPSON V370 scanner. The root length was quantified via ImageJ. Three independent biological repeats were performed.

### 4.14. Statistical Analysis

All statistical analysis was performed using a one-way ANOVA test with a significant difference via GraphPad Prism 8.0 (* *p* < 0.05; ** *p* < 0.01).

## 5. Conclusions

We aim to unravel the potential physiological function of a small signaling peptide in tomato development and adaptions to environmental stress, thus providing novel strategies for tomato cultivation and genetic breeding. To this end, we presented the comprehensive overview of putative *CEP* gene family in tomato, including their gene structure, conserved motifs and expression patterns in tissues. We also revealed that tomato *CEP* genes were differentially regulated by nitrogen form and status in roots and shoots, respectively. Synthetic tomato CEP peptides significantly promoted tomato primary root elongation via regulating NO and Ca^2+^ signaling. Additionally, we showed that the tomato CEP peptide promoted tomato root resistance to salt stress. Overall, our work would provide a very useful reference for future functional analysis CEP function in tomato, and it would also provide novel strategies to improve tomato fitness and to increase yield under adverse environments.

## Data Availability

The public transcriptome data were downloaded from Tomato Functional Genomics database, which we mentioned in “Materials and Methods”.

## Supplementary Materials

The following supporting information can be downloaded at: https://www.mdpi.com/article/10.3390/cells11192935/s1, Table S1: Summary of the identified 17 *SlCEP* genes. Table S2: Predication of N-terminal signal peptide of SlCEP proteins. Table S3: Primers for qRT-PCR analysis. Figure S1: Alignment of CEP motifs in tomato and *Arabidopsis*. Figure S2: Phylogenetic tree of full-length SlCEP proteins. Figure S3: Phylogenetic tree AtCEP and SlCEP proteins based on the CEP motif. Figure S4: Phylogenetic tree of full-length AtCEP and SlCEP proteins. Figure S5. Expression patterns of *SlCEP*s in tomato tissues.

## Abbreviations

CEP	C-TERMINALLY ENCODED PEPTIDE
CEPR1	XYLEM INTERMIXED WITH PHLOEM 1 (XIP1)/ CEP RECEPTOR 1
ROS	Reactive Oxygen Species
H_2_O_2_	Hydrogen peroxide
NO	Nitric oxide
NBT	Nitroblue tetrazolium
SNP	Sodium nitroprusside
L-NAME	L-NG-Nitro arginine methyl ester
RLK	Receptor like kinase
CEPD1	CEPD1 CEP DOWNSTREAM 1
CEPD2	CEP DOWNSTREAM 1 CEPD 2
CEPDL2	CEPD-LIKE2
CRA2	COMPACT ROOT ARCHITECTURE 2
NIN	NODULE INCEPTION
