# Supplementary material for: Systematic Annotation Reveals CEP Function in Tomato Root Development and Abiotic Stress Response
Source: Cells. 2022 Sep 20;11(19):2935. doi: 10.3390/cells11192935 (PMC9562649; doi:10.3390/cells11192935)
Supplement: Supplementary file 1 [file cells-11-02935-s001.zip › cells-1893009-supplementary.pdf]

Table S1. Summary of the identified 17 *SlCEP* genes

Gene name	Locus ID	Chromosome localization	Genomic sequence (bp)	CDS sequence (bp)	Transcript sequence (bp)	Protein sequence (AA)	Molecular weight (DA)	PI	CEP Motif (15AA)
SlCEP1	Solyc01g107570	ch01: 87387917-87388172 forward	255	255	255	85	9372.84	8.22	NVVVKNSGPSPGIGH
SlCEP2	Solyc02g065040	ch02: 34178842-34179079 reverse	237	237	237	79	8700.95	6.82	PSPGHVDGHSPGIGH
SlCEP3	Solyc02g087020	ch02:47583756-47583996 reverse	240	240	240	80	8639.74	8.81	LTEAKNSGPSPGEGH
SlCEP4	Solyc02g090590	ch02:50187107-50187356 reverse	249	249	249	83	9090.54	9.6	VDAFSSPGHSPGIGH
SlCEP5	Solyc02g090600	ch02:50192522-50193044 reverse	522	522	522	174	19058.76	9.86	NYAPKQTGNSPGIGH
									NYTPNQTGNSPGIGH
									SIDDIKAGHSPGIGH
SlCEP6	Solyc02g090610	ch02:50203465-50203768 forward	303	303	303	101	10818.55	9.6	PPPGHPDGHSPGIGH
SlCEP7	Solyc02g092890	ch02:51889669-51889942 forward	273	273	273	91	9800.43	8.71	YLGIKNSGPSPGEGH
SlCEP8	Solyc03g044180	ch03:7705647-7705914 forward	267	267	267	89	9605.11	9.6	TLGDIKDGPSPGVGH
									TLGGIKAGPSPGEGH
SlCEP9	Solyc03g044530	ch03:8919360-8919723 reverse	363	363	363	121	13256.43	6.58	DFGPTGPGHSPGIGH
SlCEP10	Solyc03g044560	ch03:9418566-9418884 reverse	318	318	318	106	12094.83	9.83	VSFDNLKDHSPGIGH
SlCEP11	Solyc03g044580	ch03:9458192-9458528 forward	336	336	336	112	12331.02	9.05	GFSPYGRGHSPGIGH
SlCEP12	Solyc07g062320	ch07:64931818-64932058 reverse	240	240	240	80	8063.94	9.16	VYGNLNIGPSPGEGH
									VYGNLNTGPSPGEGH
SlCEP13	Solyc07g062330	ch07:64935912-64936116 reverse	204	204	204	68	7365.5	6.7	IYGNLNTGPSPGEGH
SlCEP14	Solyc07g062340	ch07:64941677-64941929 reverse	252	252	252	84	8952.31	7.91	IYGNLNTKPSPGEGH
SlCEP15	Solyc07g062350	ch07:64952870-64953239 reverse	369	369	369	123	12777.46	7.85	IYGNLNTGPSPGVGH
									VYGNLNTGPSPGEGH
									VYGNLNTGPSPGEGH

SlCEP16	Solyc07g062360	ch07:64962982-64963669	reverse	687	687	687	229	23619.59	9.92	VYGNLKYDPSRGAGH
										VYHNLRSVPSPGAGH
										VYGNLRSGPSPGAGH
										VYGNLRSVPSPGAGH
SlCEP17	Solyc07g062370	ch07:64980820-64981756	reverse	936	936	936	312	32878.64	9.65	VKSESHSSPSPGEGH

Table S2. Predication of N-terminal signal peptide of SlCEP proteins

Protein	Position in sequence	Sequence	Probability	Predicated by
SlCEP1	29-30	ATEARPFI	0.6155	SignalP 5.0
SlCEP2	24-25	IVESIEGR	0.5206	SignalP 5.0
SlCEP3	24-25	CIQCRPIT	0.8301	SignalP 5.0
SlCEP4	23-24	FSQGRNLA	0.5008	SignalP 5.0
SlCEP5	52-53	FAEGRQLK		Signal-CF
SlCEP6	27-28	SIEGRHLK	0.3919	SignalP 5.0
SlCEP7	28-29	ASEARPLD	0.4857	SignalP 5.0
SlCEP8	24-25	ASEARPLN	0.7908	SignalP 5.0
SlCEP9	31-32	CVEAIRHL		Signal-CF
SlCEP10	27-28	FTEGRQLN	0.5818	SignalP 5.0
SlCEP11	26-27	TIQSIEGR	0.3915	SignalP 5.0
SlCEP12				
SlCEP13	23-24	CFEARTQL	0.5082	SignalP 5.0
SlCEP14	23-24	CFEARTQL	0.5064	SignalP 5.0
SlCEP15	23-24	CFEARTQL	0.4620	SignalP 5.0
SlCEP16	23-24	CFEAKTQS	0.4772	SignalP 5.0
SlCEP17	21-22	SFEARKQF	0.7030	SignalP 5.0

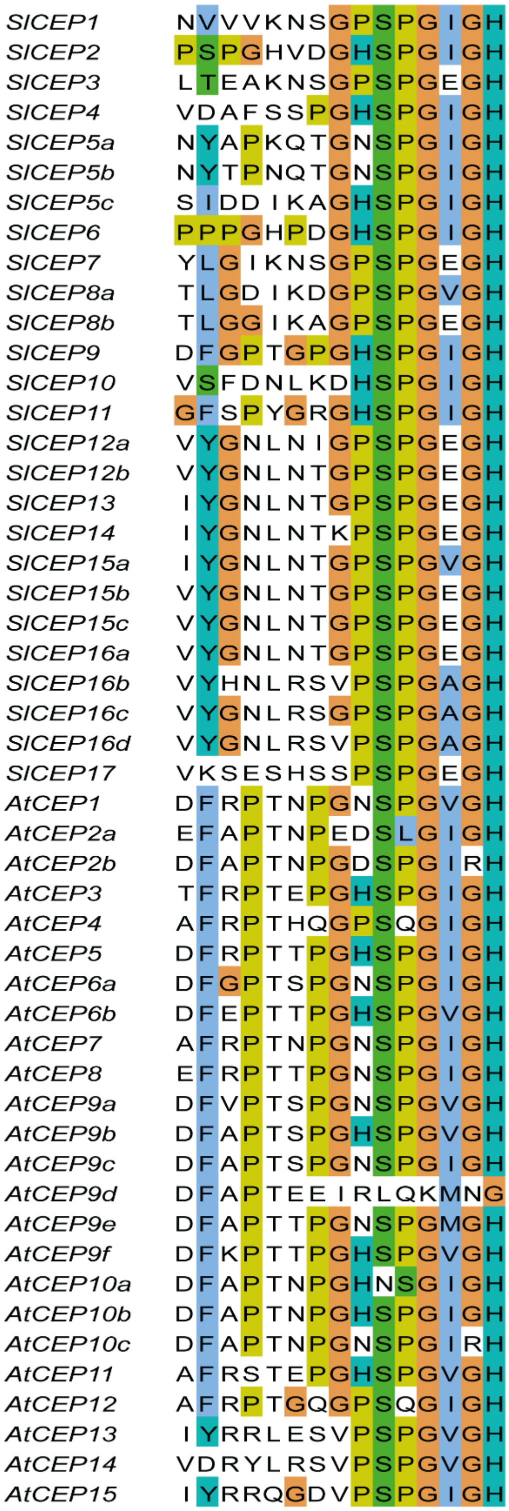

Figure S1. Alignment of CEP motifs in tomato and *Arabidopsis*.

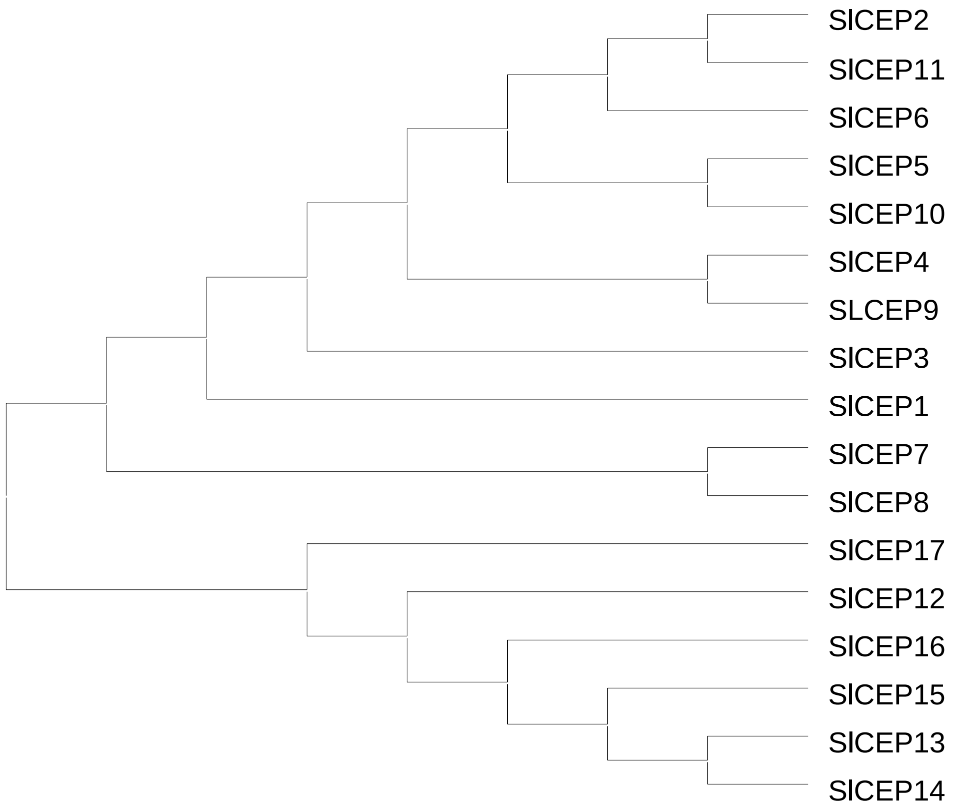

Figure S2. Phylogenetic tree of full-length SlCEP proteins. The phylogenetic tree was build based on the full-length sequences of SlCEP proteins with 1000 bootstrap replicates.

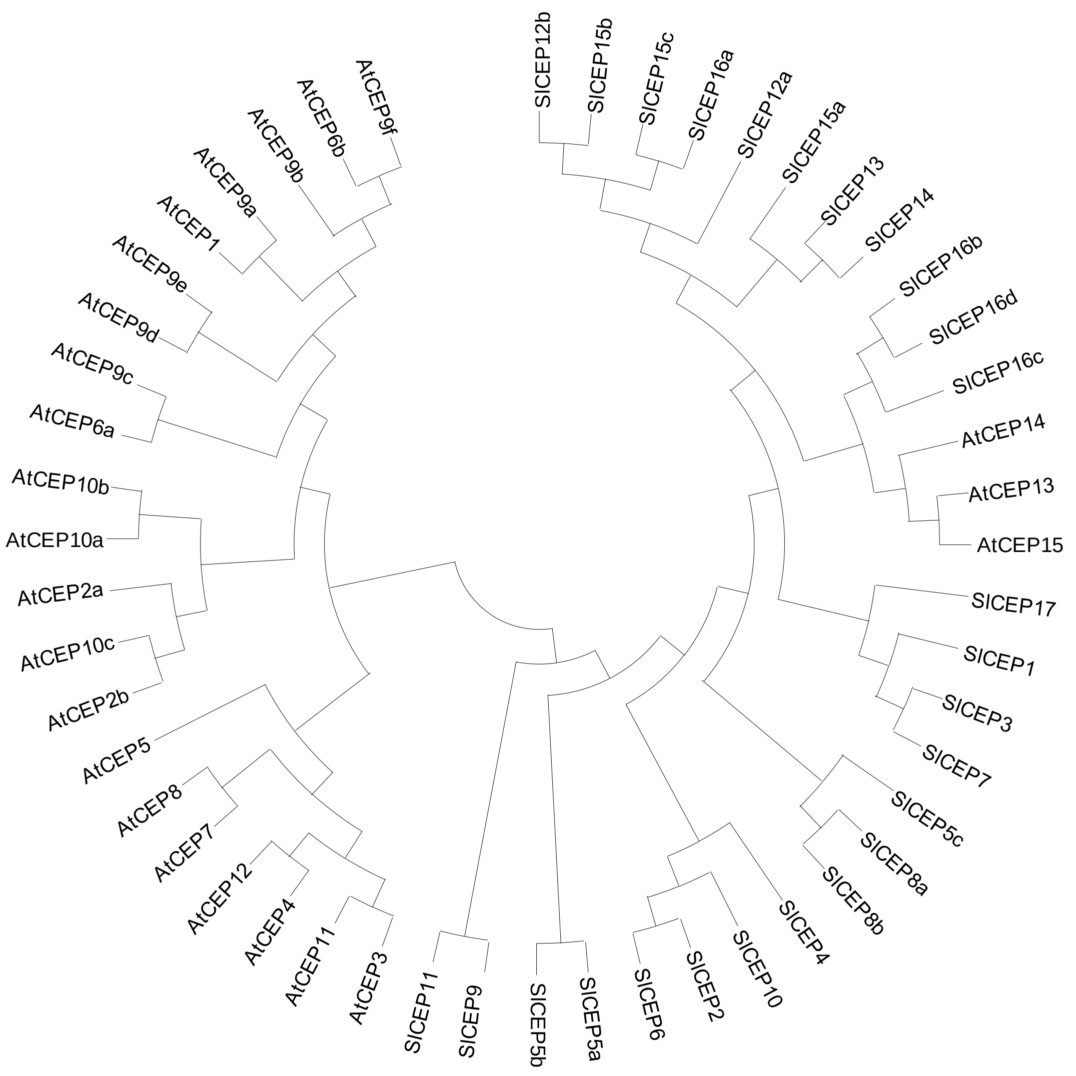

Figure S3. Phylogenetic tree of AtCEP and SlCEP proteins based on the CEP motifs. The phylogenetic tree was build based on the CEP motifs of SlCEP and AtCEP proteins with 1000 bootstrap replicates.

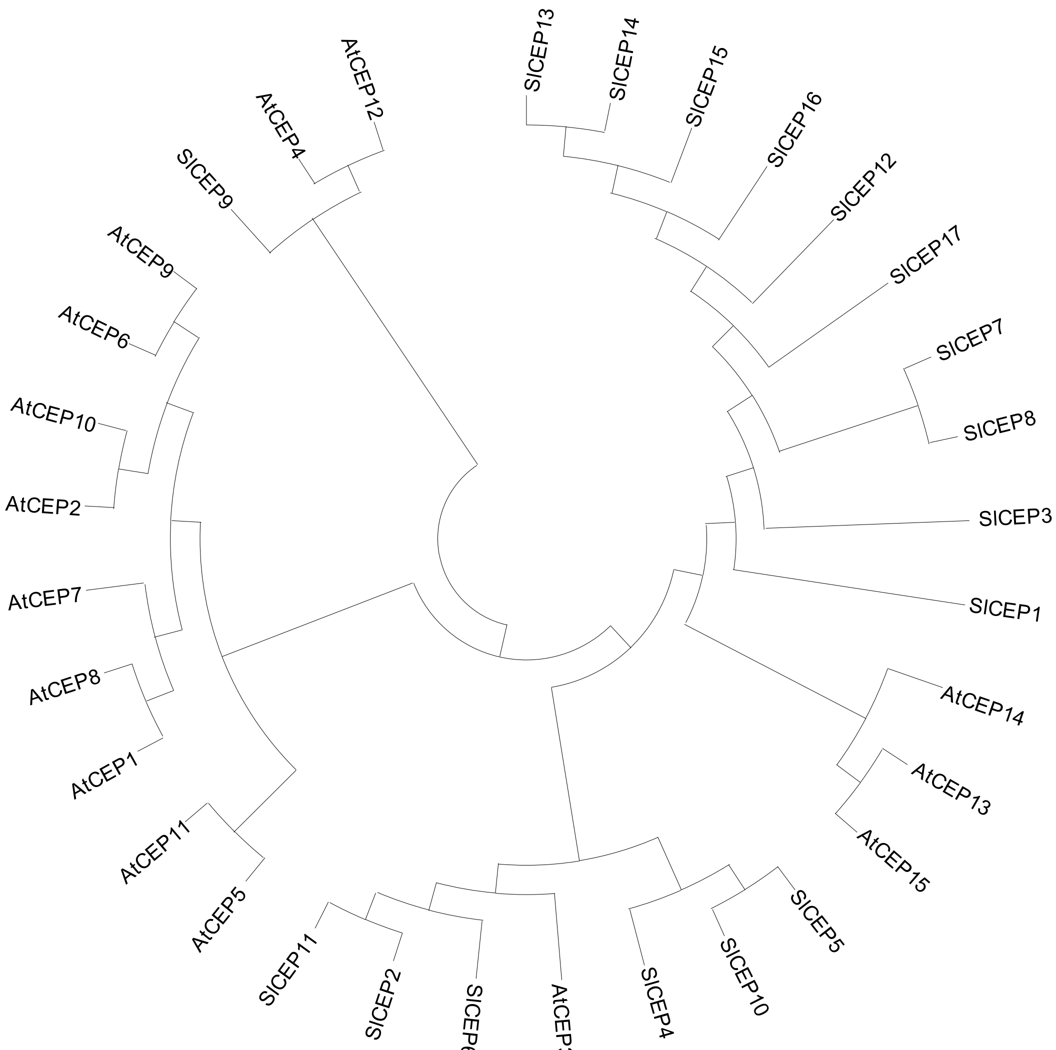

Figure S4. Phylogenetic tree of full-length AtCEP and SlCEP proteins. The phylogenetic tree was build based on the full length of SlCEP and AtCEP proteins with 1000 bootstrap replicates.

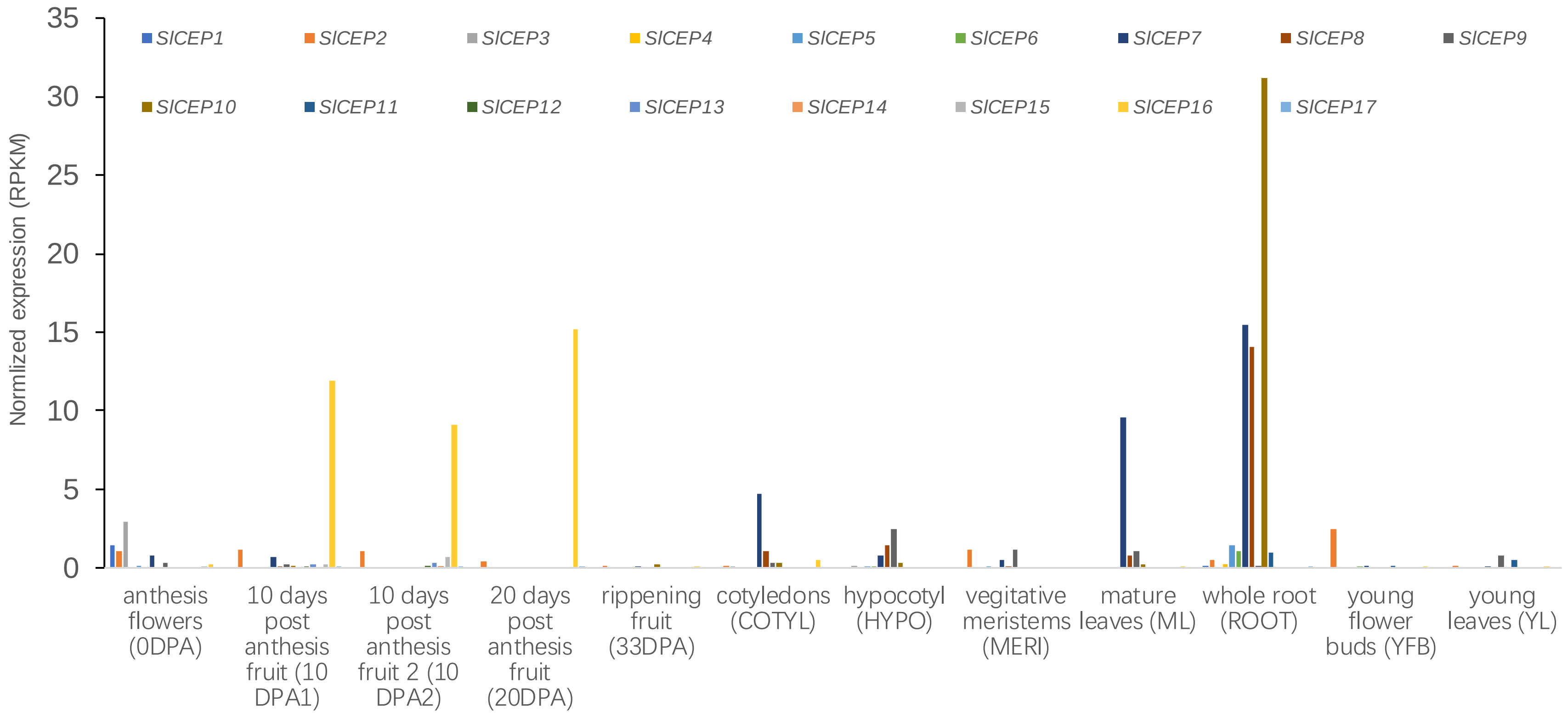

Figure S5. Expression levels of *SlCEP*s in tomato tissues. The expression data was extracted from a transcriptome analysis of various tissues in wild species *S. pimpinellifolium*, LA1589.

Table S3 Primers used for qRT-PCR analysis

SlCEP1F	ATGGCTCGAGTTATCATTCA	***SlCEP10F***	TATGCATTTACTTTCCTTCT
SlCEP1R	AGGACCGGAGTTTTTCACCA	***SlCEP10R***	TTCTTCATACCTTTGCTTTG
SlCEP2F	TCCTTGCTATCTTTGTCCTT	***SlCEP11F***	TTCAATCTATCGAGGGACGA
SlCEP2R	CATTGTTTTGAACAGTGAAT	***SlCEP11R***	CGTCAAATGTTCCCTTCTTT
SlCEP3F	ATGAAAATGGGCACAACAAA	***SlCEP12F***	ATGTTTATGGCAACTTGAAT
SlCEP3R	AAGTTGATGAACTTTCATGC	***SlCEP12R***	AAACATGACCCATTCTAGGA
SlCEP4F	ATGGCTAAGTTTTATACCGT	***SlCEP13F***	TATGTTAGTTTTCGTTGCAC
SlCEP4R	TTTATCTTCCTTTTCTTCGA	***SlCEP13R***	TGTCCTTCACCAGGACTAGG
SlCEP5F	GCTGAGGGAAGACAACTGAA	***SlCEP14F***	ACTATGTTAGTTTTCGTTGC
SlCEP5R	TCCGGTTTGCTTTGGTGCAT	***SlCEP14R***	TCCTTCTCCAGGACTAGGTT
SlCEP6F	AGGGAAGACATTTGAAGTTG	***SlCEP15F***	TTGCACCATGTTTTGAAGCA
SlCEP6R	TCTGAACCAGACTAGTTGGC	***SlCEP15R***	GAGGTGGGCCAAAATGTCCG
SlCEP7F	GTAAATGCAAGAGCTTCATG	***SlCEP16F***	CTTGCTTCTTCGGCCCCATA
SlCEP7R	TAAGGCCATTCTTGATAGCA	***SlCEP16R***	ACCAGCTCCAATACTAAGGC
SlCEP8F	ATGGTGAGATTTTCTTATTT	***SlCEP17F***	AATTTAGCAAAGAAGGTAAC
SlCEP8R	CTTAATATCACCCAATGTTA	***SlCEP17R***	GACTCGAGACTGATTTCAAA
SlCEP9F	ATGGTTATTGTAACAAACAC	***SlActinF***	CTCAGTCAGGAGAACAGGGT
SlCEP9R	TGATACGAAATTTCCACAC	***SlActinR***	GCCTCCAGCCTTGTTGTAAA
